# Fault Isolation Filter for Networked Control System with Event-Triggered Sampling Scheme

**DOI:** 10.3390/s110100557

**Published:** 2011-01-07

**Authors:** Shanbin Li, Dominique Sauter, Bugong Xu

**Affiliations:** 1 College of Automation Science and Engineering, South China University of Technology, Guangzhou 510641, China; 2 Nancy University, CRAN-CNRS UMR 7039, BP239, 54506 Vandoeuvre Cedex, France

**Keywords:** networked control system, fault isolation filter, event-triggered sampling, send-on-delta

## Abstract

In this paper, the sensor data is transmitted only when the absolute value of difference between the current sensor value and the previously transmitted one is greater than the given threshold value. Based on this send-on-delta scheme which is one of the event-triggered sampling strategies, a modified fault isolation filter for a discrete-time networked control system with multiple faults is then implemented by a particular form of the Kalman filter. The proposed fault isolation filter improves the resource utilization with graceful fault estimation performance degradation. An illustrative example is given to show the efficiency of the proposed method.

## 1. Introduction

Over recent years, fault diagnosis for networked control system (NCS) using the mode-based analytical redundancy method have received significant attention. In [1,2], the overviews of main ideas and results on fault diagnosis of NCS are given, including the fundamentals of fault diagnosis for NCS with information scheduling, fault diagnosis approaches based on the simplified time-delayed system models and the quasi T-S fuzzy model, and fault diagnosis for linear and nonlinear NCS with long time-delay. However, most of the available results make use of time-triggered state estimation techniques by sampling the output of plant at an essentially equidistant time instant. Because the sampling period is determined according to the worst case operation conditions that rarely occur, the time-triggered sampling leads to a conservative usage of the communication bandwidth.

On the other hand, recent advances in computing and communication technologies enable the wireless networks (e.g., Bluetooth, wirelessHART and ZigBee) to rapidly replace wired networks in many applications, including industrial control and monitoring, home automation and consumer electronics, security and military sensing, and health monitoring [3,4]. Though the wireless channels are easier and cheaper to deploy and avoid cumbersome cabling, they also pose serious resource constraints. Therefore, applying the time-triggered sampling method to the wireless NCS may have some negative effects on the estimation and control performance of system, such as wasting the scarce communication resource and further shortening the lifetime of overall system. Since the event-triggered sampling strategies present a number of potential advantages for NCS, such as clock-free operation, less traffic requirement, and better resource utilization, they have been regarded as the possible and important alternatives to the time-triggered sampling.

Until now, numerous event-triggered sampling concepts have been proposed in the literature, such as send-on-delta sampling [5,6], level-crossing sampling [7], deadband sampling [8], Lebesgue sampling [9], send-on-area sampling [10], error energy sampling [11], self-triggered sampling [12], *etc*. Although these schemes have different terminologies, the same attribute is that the signal is sampled only when an *a priori* defined events occurs in the data monitored by sensors. For instance, the studies in [5–9] are concerned with the same sampling criterion where the event is defined as that the difference Δ between the current sensor value and the last transmitted one is greater than a given threshold. While in [10] and [11], the event is that the integral and energy of Δ is greater than a given threshold, respectively. Because of inherent distinctive benefits, the so-called event-triggered state estimation and event-based control for NCS with event-triggered sampling schemes have gained increasing attention. In this paper, however, we focus our attention on the event-triggered state estimation for the purpose of fault diagnosis. In relation to a parallel line of research on the event-based control, we refer the readers to the literature, e.g., [12,13].

In the context of state estimation, although the time-triggered state estimation over networks with network-induced effects taken into account have made great progress (see, e.g., [14–17]), research on the event-triggered state estimation is relatively lacking apart from several works [6,10,18–26]. It is well known that utilizing more sensors can potentially improve the performance of the estimation algorithms. However, using too many sensors can in turn create bottlenecks in the communication resource when these sensors compete for bandwidth. As a result, the studies in [18–21] explore the tradeoff between communication and estimation performance. Rather than sending every raw measurement to the remote estimator via network, a so-called controlled communication policy was adapted, which firstly obtain the local estimate *x̃_k|k_* from the raw sensor measurements and then compare *x̃_k|k_* with the remote estimate to decide whether or not it is worth sending data *x̃_k|k_*. Also, Reference [21] proposes an optimal communication policy by dynamic programming and value iteration to minimize a long-term average cost function, which is related to the difference between the local and remote estimate. Based on the send-on-delta method, Reference [6] proposes a modified Kalman filter where computed output with increased measurement noise covariance is used when there is no sensor data transmission. The authors also discuss how to choose the threshold which is a trade-off parameter between the sensor data transmission rate and the estimation performance. Reference [22] extends the previous work [6] to address how to determine the measurement value at a sensor node if it does not send data. To avoid the inability of send-on-delta method in detecting the signal oscillations or steady-state error, Reference [10] proposes a novel scheme called send-on-area and then formulates a networked estimator based on Kalman filter to estimate the states of the system. More recently, Reference [23] proposes a networked estimator for event-triggered sampling systems with packet dropouts. Reference [24] develops an event-triggered estimator which is updated both when an event occurs with a received measurement sample, as well as at sampling instants synchronous in time without receiving a measurement sample. However, to the authors’ knowledge, fault diagnosis of networked control systems making use of the event-triggered state estimation method has not been addressed, which motivates the current study of this paper.

In this paper, we show our attention on the implementation problem of a modified fault isolation filter (FIF) for NCS with event-triggered sampling and multiple faults. By the send-on-delta scheme which is one of the event-triggered sampling strategies, it means that the sensor data is transmitted only when the absolute value of difference between the current sensor value and the previously transmitted one is greater than the given threshold value. Based on this scheme, a modified FIF for a discrete-time NCS with multiple faults is then implemented by a particular form of the Kalman filter. The rest of this paper is organized as follows. The modelling of NCS with event-triggered sampling scheme is presented in Section 2. A modified FIF is proposed in Section 3. An illustrative example is presented in Section 4 to show the effectiveness of the result. The paper is concluded in Section 5.

**Notations:** In what follows, if not explicitly stated, matrices are assumed to have compatible dimensions. *Z*_+_ denotes the set of nonnegative integer numbers. *ℛ^n^* and *ℛ^n×m^* are, respectively, the *n*-dimensional Euclidean space and the set of *n × m* real matrices. *A^T^* denotes the transpose matrix or vector *A*. *A*^−1^ and *A*^+^ represent the inverse and pseudo-inverse of *A*, respectively. diag(*a*_1_,…,*a_n_*) refers to an *n × n* diagonal matrix with *a_i_* as its *i*th diagonal entry. rank(*A*) stands for the rank operator of matrix *A*. *ℰ*(*x*) represents the mathematical expectation of random variable *x*. *x ∼ 𝒩*(μ, Σ) means that the random vector satisfies the normal distribution with mean value μ and covariance matrix Σ. *𝒫*(*x|y*) means the conditional probability distribution of *x* given *y. Sign* function is defined as $\mathrm{sign}(x)=\{\begin{array}{ll} 1, & x\geq 0;\\ -1, & x<0. \end{array}$

## 2. NCS with Event-Triggered Sampling Scheme

**Figure 1.**
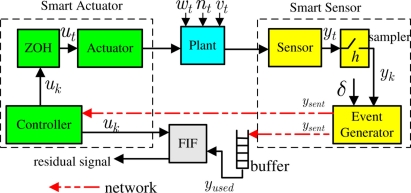
The architecture of NCS with event-triggered sampling.

The architecture of NCS with event-sampling discussed in this paper is shown in Figure 1, where the closed-loop system consists of a plant with smart sensors and actuators, a remote FIF and a wireless network channel. The controller, FIF and actuator are assumed to be logically integrated. Thus, control commands do not need to experience any wireless transmission. This configuration represents a system, e.g., wireless sensor/actuator system, where actuation is inexpensive but sensor measurements are transmitted to the controller or FIF by sensors with a limited energy.

Since the event generator, the controller and the FIF have to be implemented on smart sensors and actuators by means of digital hardware, a discrete-time plant model is considered as the alterative to the continuous plant together with a zero-order hold and a sampler. The state evolution and sensor measurement equation are given as follows, respectively:

(1) $$\{\begin{array}{ll} x_{k+1} & ={\mathrm{Ax}}_{k}+{\mathrm{Bu}}_{k}+{\mathrm{Fn}}_{k}+w_{k}\\ \;\;\;\;y_{k} & ={\mathrm{Cx}}_{k}+v_{k} \end{array}$$

where *x_k_* ∈ *ℛ^n^* is the state vector, *u_k_* is the known manipulated input, $F=[\begin{array}{cccc} f_{1} & f_{2} & \ldots & f_{q} \end{array}]\in {ℛ}^{n\times q}$ is the fault distribution matrix, *n_k_* ∈ *ℛ^q^* is the fault vector, and $y_{k}={\left[\begin{array}{lllll} y_{k}^{1} & \ldots & y_{k}^{i} & \ldots & y_{k}^{m} \end{array}\right]}^{T}\in {ℛ}^{m}$ the output observation vector of sensors. We assume that the initial state vector *x*_0_, process noise *w_k_* and measurement noise *v_k_* are uncorrelated, zero mean white Gaussian random processes with *x*_0_ *˜ 𝒩* (μ_0_, Σ_0_), *w_k_ ˜ 𝒩*(0, *W*) *v_k_ ˜ 𝒩*(0, *R*), where Σ_0_, *W* and *R* are symmetric, positive definite matrices.

**Figure 2.**
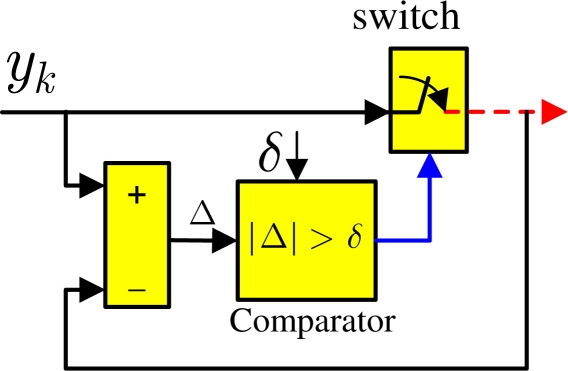
The architecture of event generator.

As shown in Figure 1, the smart sensor numbered *i* has a sampler which regularly samples the sensor measurement with period *h* and an event generator to decide whether or not to send new sensor measurement through the network. The event generator therein, also known as send-on-delta scheme, is illustrated in Figure 2 where the sensor data $y_{k}^{i}$ is transmitted only when the absolute value of difference between the current sensor value $y_{k}^{i}$ and the previously transmitted one $y_{\mathrm{sent}}^{i}$ is greater than the given threshold value δ*_i_*, namely

(2) $$\left|y_{k}^{i}-y_{\mathrm{sent}}^{i}\right|>\delta_{i}$$

where δ*_i_* is simply determined according to *x*-fold of the maximal amplitude of $y_{k}^{i}$.

Obviously, all the transmitted measurements $y_{\mathrm{sent}}^{i}$ are the event-triggered samplers which are subsequences of the raw measurement $y_{k}^{i}$. For instance, if the time instant when the previous event occurs is denoted as *k_j_* ∈ *Z*_+_, the event-triggered sampling condition (2) is further formulated as

(3) $$\left|y_{k}^{i}-y_{k_{j}}^{i}\right|>\delta_{i},k\in \left[k_{j},k_{j+1}\right),k_{j}\in Z_{+}$$

**Figure 3.**
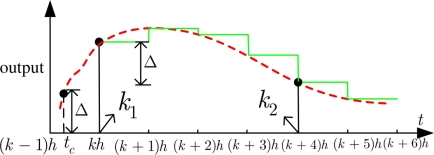
Send-on-delta sampling in discrete-time case.

However, the time instant *k_j_* (*j* = 1, 2....) can not be precisely determined because of the sampler, which is significantly different from the continuous one introduced in e.g., [5,6]. As shown in Figure 3, the dash line and real line represent the sensor measurement in the continuous time and discrete time, respectively. In the continuous time case, the time *t_c_* when an event occurs can be exactly known since the measurement of plant is continuously updated by the sensor. While in the discrete time, the events have to be generated at the subsequent discrete time steps, e.g., *k*_1_ equivalent to *kh*, *k*_2_ equivalent to (*k* + 4)*h*, since the measurement is only updated at some discrete time instants and remains constant in the inter-sampling interval. Although producing the unsent measurements, e.g., the sample at the time instant (*k* + 1)*h*, wastes a bit of computing resource, the unsent measurements in turn save a lot of communication resources. In a sense of improving the resource utilization, applying the event-triggered sampling schemes to discrete-time plant is also meaningful. The reason is that wireless communication consumes more energy than information processing. As noted in [5], a sensor node can execute 3,000 instructions for the same energy cost of sending a single bit at the distance of 100 meters by radio.

**Figure 4.**
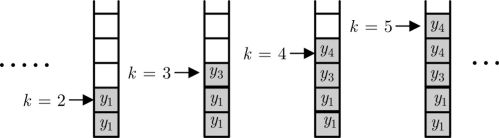
The configuration of the buffer.

In the sequel, we further assume that all sensor measurements are time-stamped and the network is communication link without packet losses and time delays. For the purpose of reducing communication, only parts of the raw measurements will be communicated to the remote FIF by the send-on-delta scheme. Through the communication link, all the event-triggered samplers ${\left\{y_{k_{j}}^{i}\right\}}_{j=1}^{\infty }$ are then stored in an infinite buffer. If sensor measurement $y_{k}^{i}$ does not yet arrive at the buffer at time instant *k*, it means that the current value $y_{k}^{i}$ has not significantly changed in contrast to the previous event-triggered sampler $y_{k_{j}}^{i}$. In this case, the previous buffer value $z_{k-1}^{i}$ whose value is equivalent to $y_{k_{j}}^{i}$ will be stored in the *k*-slot of the buffer, as illustrated in Figure 4.

Furthermore, the arrival of the measurement $y_{k}^{i}$ at time *k* is defined as a binary variable $\theta_{k}^{i}$, namely $\theta_{k}^{i}=1$ when $y_{k}^{i}$ arrives at the buffer at time instant *k*, otherwise $\theta_{k}^{i}=0$. Rather than considering the design problem where only the probabilities of $\theta_{k}^{i}$ at each time instant is known and the research interest lies in studying the effect of loss and delay probabilities, we address the implementation problem where the value of $\theta_{k}^{i}$ at each time instant is known in advance. The last received value of *i*-th sensor output at time instant *k_j_* is denoted as $y_{k_{j}}^{i}$. If there is no sensor data received for *k > k_j_*, the estimator node considers that the measurement value of the *i*-th sensor output $y_{k}^{i}$ is still equal to $y_{k_{j}}^{i}$, but the measurement noise is increased from $\upsilon_{k}^{i}$ to ${\bar{\upsilon }}_{k}^{i}=\upsilon_{k}^{i}+\Delta_{k,kj}^{i}$ where $\Delta_{k,k_{j}}^{i}=y_{k_{j}}^{i}-y_{k}^{i}$ satisfies $\left|\Delta_{k,k_{j}}^{i}\right|\leq \delta_{i}$.

From the existing literature [6,22], the assumption that $\Delta_{k,k_{j}}^{i}$ has a uniform distribution with zero mean and a variance $\delta_{i}^{2}/3$ is valid only if the measurement covariance *R >* δ^2^. Otherwise, the mean and variance of Δ*_i_*(*k*, *k_j_*) is $\mathrm{sign}(y_{k_{j}}^{i}-y_{k_{j}-1}^{i})*\delta_{i}/2$ and *δ_i_*/12 respectively. Thus, the measurements $z_{k}^{i}$ which the FIF will use for fault diagnosis are formulated as

(4) $$z_{k}^{i}=\theta_{k}^{i}y_{k}^{i}+\left(1-\theta_{k}^{i}\right)y_{k_{j}}^{i}$$

and the output noise $\upsilon_{k}^{i}$ of the smart sensor numbered *i* is defined as:

(5) $$𝒫\left(v_{k}^{i}|\theta_{k}^{i}\right)=\{\begin{array}{ll} 𝒩\left(0,R_{i,i}\right), & \theta_{k}^{i}=1\\ 𝒩\left(0,R_{i,i}+\delta_{i}^{2}/3\right), & \theta_{k}^{i}=0 \end{array}$$

if $R_{i,i}>\delta_{i}^{2}$. Otherwise, the output noise $\upsilon_{k}^{i}$ is defined as:

(6) $$𝒫\left(v_{k}^{i}|\theta_{k}^{i}\right)=\{\begin{array}{ll} 𝒩\left(0,R_{i,i}\right), & \theta_{k}^{i}=1\\ 𝒩\left(\mathrm{sign}\left(y_{k_{j}}^{i}-y_{k_{j}-1}^{i}\right)*\delta_{i}/2,R_{i,i}+\delta_{i}^{2}/12\right), & \theta_{k}^{i}=0 \end{array}$$

Moreover, the following selector is designed to flexibly determine whether (5) or (6) is applied to the FIF:

(7) $$\mathrm{Selector}=\{\begin{array}{ll} (5), & \left|y_{k_{j}}^{i}-y_{k_{j}-1}^{i}\right|\leq \varepsilon_{i}\\ (6), & \text{otherwise} \end{array}$$

where *ε_i_* > 0 is a sufficiently small threshold.

By the selector (7) and compensating some values for the unsent measurements, we can also regularly implement some existing fault isolation filter algorithms, e.g., [27] to NCS with event-triggered sampling even though the measurements are transmitted irregularly.

## 3. Modified Fault Isolation Filter

Fault isolation filter, a special dynamic observer which generates the directional residuals in response to a particular fault, is an attractive way for enhancing the fault isolability [28,29]. It was first developed by Beard [28] and [29] and later revisited by Massoumnia [30] in the geometric framework and by White and Speyer [31] and Park and Rizzoni [32] in the context of eigenstructure assignment. Further improvements were suggested by Liu and Si [33], and Keller [27]. For linear continuous time-invariant system, Liu and Si [33] have proposed a fault isolation filter such that faults can be asymptotically detected and isolated. To guarantee that the *i*th component of the output residual is decoupled from all but the *i*th fault, the columns of the fault detectability matrix are assigned as the eigenvectors of the filter’s transition matrix with a set of fixed eigenvalues. Keller extended this approach to discrete-time stochastic linear systems. A new fault isolation filter has been developed to isolate *q* faults with at least *q* output measurements. More recently, Reference [34] addressed the fault detection problem for a class of linear networked control systems by extending the FIF proposed in [27].

In this section, we further construct a modified Keller’s FIF to detect and isolate the multiple faults in NCS with event-triggered sampling.

Recalling the definitions of fault detectability indexes and matrice introduced in [27]:

**Definition 1.** *The linear stochastic system (1) is said to have fault detectability indexes ρ* = {*ρ*_1_, *ρ*_2_, …, *ρ_q_*} *if ρ_i_* = min{*ν*: *CA*^ν−1^ *f_i_* ≠ 0, *ν* = 1,2,…}

**Definition 2.** *If the linear stochastic system (1) has finite fault detectability indexes, the fault detectability matrix D is defined as:*

(8) D=CΨ

*with*

$$\Psi =\left[\begin{array}{ccccc} A^{\rho_{1}-1}f_{1} & \cdots & A^{\rho_{i}-1}f_{i} & \cdots & A^{\rho_{q}-1}f_{q} \end{array}\right]$$

Now, the following filter is presented as the residual generator of discrete-time plant (1):

(9) $$\{\begin{array}{ll} {\hat{x}}_{k+1} & =A{\hat{x}}_{k}+{\mathrm{Bu}}_{k}+K_{k}\left(y_{k}-C{\hat{x}}_{k}\right)\\ \alpha_{k} & =L_{k}\left(y_{k}-C{\hat{x}}_{k}\right) \end{array}$$

where *x̂_k_* is the state of the filter, *α_k_* the residual generator or the fault indicator. Filter gain *K_k_* ∈ *ℛ^n×m^* and projector *L_k_* ∈ *ℛ^q×m^* are unknown matrices to be found for the solution of the fault detection and isolation problem.

From Equations (1) and (9), the state estimation error *e_k_* = *x_k_ − x̂_k_* and the output of the filter *α_k_* propagate as

(10) $$\{\begin{array}{ll} e_{k+1} & =\left(A-K_{k}C\right)e_{k}+{\mathrm{Fn}}_{k}-K_{k}v_{k}+w_{k}\\ \alpha_{k} & =L_{k}\left({\mathrm{Ce}}_{k}+v_{k}\right) \end{array}$$

Let *G_nα_*(*z*) be the transfer function from *n_k_* to the output residual *α_k_*. Then the following theorem is presented to design *K_k_* and *L_k_* such that

(11) $$\begin{array}{ll} G_{n\alpha }(z) & =L_{k}C{(zI-\left(A-K_{k}C\right))}^{-1}F\\ & =\text{diag}\left\{z^{-\rho_{1}},\ldots ,z^{-\rho_{q}}\right\} \end{array}$$

which ensures the isolation of multiple faults.

**Theorem 1.** *Under the condition* rank(*D*) *= q, the solutions of (11) can be parameterized as K_k_* = ωΠ+*K̄_k_*Σ, *L_k_* = Π, *with* Σ = *β*(*I − D*Π), Π = *D*^+^, ω = *A*Ψ *and D* = *C*Ψ, *where K̄_k_* ∈ *ℛ^n×m−q^ is the free parameters to be designed, D^+^ is the pseudo-inverse of D and β is an arbitrary matrix chosen so that* rank(Σ) = *m* − *q*

From Theorem 1, the fault isolation filter (9) is rewritten from the free parameter *K̄_k_* as

(12) $$\{\begin{array}{ll} {\hat{x}}_{k+1} & =A{\hat{x}}_{k}+{\mathrm{Bu}}_{k}+{\omega \alpha }_{k}+{\bar{K}}_{k}\gamma_{k}\\ \alpha_{k} & =\Pi \left(y_{k}-C{\hat{x}}_{k}\right)\\ \gamma_{k} & =\Sigma \left(y_{k}-C{\hat{x}}_{k}\right) \end{array}$$

where *α_k_* is a deadbeat filter of fault *n_k_* and given by:

(13) $$\alpha_{k}=\Pi {\overset{⌣}{\alpha }}_{k}+{\left[\begin{array}{lllll} n_{k-\rho_{1}}^{1T} & \ldots & n_{k-\rho_{i}}^{iT} & \ldots & n_{k-\rho_{q}}^{qT} \end{array}\right]}^{T}$$

The fault $n_{k-\rho i}^{i}$ of detectability index *ρ_i_* directly affects the reduced output residual *α_k_* with a time delay equals to its detectability index. *ᾰ_k_* is the fault indicator signal without faults and propagates from the fault-free state estimation error *ē_k_* = *x̃_k_* − *x̂_k_* as

(14) $$\{\begin{array}{ll} {\bar{e}}_{k+1} & =\left(\bar{A}-{\bar{K}}_{k}\bar{C}\right){\bar{e}}_{k}-{\bar{K}}_{k}v_{k}+w_{k}\\ {\overset{⌣}{\alpha }}_{k} & =\Pi \left(C{\bar{e}}_{k}+v_{k}\right) \end{array}$$

where *Ā* = *A* − ωΠ*C*, *C̄* = Σ*C* and *x̃_k_* is the fault-free state.

From Equation (14), the following theorem is then proposed to design the free parameter *K̄_k_* which minimizes the trace of the fault-free state estimation error covariance matrix ${\bar{P}}_{k+1}=ℰ\{{\bar{e}}_{k+1}{\bar{e}}_{k+1}^{T}\}$.

**Theorem 2.** *The proposed fault isolation filter described by the following relations:*

(15) $$\{\begin{array}{ll} {\hat{x}}_{k+1} & =A{\hat{x}}_{k}+{\mathrm{Bu}}_{k}+{\omega \alpha }_{k}+{\bar{K}}_{k}\gamma_{k}\\ \alpha_{k} & =\Pi \left(y_{k}-C{\hat{x}}_{k}\right)\\ \gamma_{k} & =\Sigma \left(y_{k}-C{\hat{x}}_{k}\right)\\ {\bar{P}}_{k+1} & =\left(\bar{A}-{\bar{K}}_{k}\bar{C}\right){\bar{P}}_{k}{\left(\bar{A}-{\bar{K}}_{k}\bar{C}\right)}^{T}+{\bar{K}}_{k}\bar{V}{\bar{K}}_{k}^{T}+\bar{W}+\omega \Pi R\Sigma^{T}{\bar{K}}_{k}^{T}+{\bar{K}}_{k}\Sigma R\Pi^{T}\omega^{T}\\ {\bar{K}}_{k} & =\left(\bar{A}{\bar{P}}_{k}{\bar{C}}^{T}-\omega \Pi R\Sigma^{T}\right){\left(\bar{C}{\bar{P}}_{k}{\bar{C}}^{T}+\bar{V}\right)}^{-1} \end{array}$$

*with V̄* = Σ*R*Σ*^T^*, *W̄* = *W* + ωΠ*R*Π*^T^*ω*^T^*.

Based on Theorem 2 and the measurement noise shown in Equations (5) and (6), the modified FIF for NCS with event-triggered sampling is proposed as follows:

**Algorithm 1.** Initialization

*set x*_0_, *P*_0_, *δ_i_*, *ε_i_* *if ith sensor data are received* $\left(\theta_{k}^{i}=1\right)$ $\bar{R}(i,i)=R(i,i),z_{k}^{i}=y_{k}^{i},\eta_{k}^{i}=0$ *else if* $\left|y_{k_{j}}^{i}-y_{k_{j}-1}^{i}\right|\leq \varepsilon_{i}\%\;\varepsilon_{i}$ *is a sufficiently small sclar* $\bar{R}(i,i)=R(i,i)+\delta_{i}^{2}/3,\eta_{k}^{i}=0$ else $\bar{R}(i,i)=R(i,i)+\delta_{i}^{2}/12,\eta_{k}^{i}=\mathrm{sign}\;\left(y_{k_{j}}^{i}-y_{k_{j}-1}^{i}\right)*\delta_{i}^{2}/2$ *end if* $$\begin{array}{l} z_{k}={\left[\begin{array}{lllll} z_{k}^{1} & \ldots & z_{k}^{i} & \ldots & z_{k}^{m} \end{array}\right]}^{T}\\ y_{k}={\left[\begin{array}{lllll} y_{k}^{1} & \ldots & y_{k}^{i} & \ldots & y_{k}^{m} \end{array}\right]}^{T}\\ \eta_{k}={\left[\begin{array}{lllll} \eta_{k}^{1} & \ldots & \eta_{k}^{i} & \ldots & \eta_{k}^{m} \end{array}\right]}^{T}\\ z_{k}=y_{k}+\eta_{k} \end{array}$$ *Computing* (16) $$\{\begin{array}{ll} {\hat{x}}_{k+1} & =A{\hat{x}}_{k}+{\mathrm{Bu}}_{k}+{\omega \alpha }_{k}+{\bar{K}}_{k}\gamma_{k}\\ \alpha_{k} & =\Pi \left(z_{k}-C{\hat{x}}_{k}\right)\\ \gamma_{k} & =\Sigma \left(z_{k}-C{\hat{x}}_{k}\right)\\ {\bar{P}}_{k+1} & =\left(\bar{A}-{\bar{K}}_{k}\bar{C}\right){\bar{P}}_{k}{\left(\bar{A}-{\bar{K}}_{k}\bar{C}\right)}^{T}+{\bar{K}}_{k}\bar{V}{\bar{K}}_{k}^{T}+\bar{W}+\omega \Pi \bar{R}\Sigma^{T}{\bar{K}}_{k}^{T}+{\bar{K}}_{k}\Sigma \bar{R}\Pi^{T}\omega^{T}\\ {\bar{K}}_{k} & =\left(\bar{A}{\bar{P}}_{k}{\bar{C}}^{T}-\omega \Pi \bar{R}\Sigma^{T}\right){\left(\bar{C}{\bar{P}}_{k}{\bar{C}}^{T}+\bar{V}\right)}^{-1} \end{array}$$ *with V̄* = Σ*R̄*Σ*^T^*, *W̄* = *W* + ωΠ*R̄*Π*^T^*ω*^T^*.

## 4. Illustrative Example

In this section, we will present an example to illustrate the implementation approach proposed in this paper. The modified example is borrowed from [27] described by Equation (1), where the parameters are as follows:

$$\begin{array}{l} A=\left[\begin{array}{cccc} 0.2 & 1 & 0 & 0.2\\ 0 & 0.5 & 1 & 0.4\\ 0 & 0 & 0.8 & 1\\ 0 & 0 & 0 & 0.3 \end{array}\right],B=\left[\begin{array}{cc} 0 & -1\\ -1 & 1\\ 1 & 0\\ -1 & 1 \end{array}\right],F=\left[\begin{array}{cc} -1 & 1\\ 1 & 0\\ 0 & -1\\ 1 & 1 \end{array}\right],C=\left[\begin{array}{cccc} 0 & 1 & 0 & 0\\ 0 & 0 & 1 & 0\\ 0 & 0 & 0 & 1 \end{array}\right],\\ W=\left[\begin{array}{cccc} 0.9 & 0 & 0 & 0\\ 0 & 0 & 0 & 0\\ 0 & 0 & 0.5 & 0\\ 0 & 0 & 0 & 0.3 \end{array}\right],R=\left[\begin{array}{ccc} 0.3 & 0 & 0\\ 0 & 0.1 & 0\\ 0 & 0 & 0.5 \end{array}\right] \end{array}$$

The faults associated with the first and second column of the fault distribution matrix *F* occur at time instant *r*_1_ = 10 with $n_{k}^{1}=5\text{sin}(0,2k)$ and *r*_2_ = 30 with $n_{k}^{2}=15$, respectively.

By Theorem 1, we have *D* = *C*Ψ and Ψ = *F* since rank(*CF*) = *q* (*ρ*_1_ = 1, *ρ*_2_ = 1). The parametrization of the FIF’s gain *K_k_* and projector *L_k_* is then given by

$$\Sigma =\left[\begin{array}{ccc} -0.333 & 0.333 & 0.333 \end{array}\right],\Pi =\left[\begin{array}{ccc} 0.6667 & 0.3333 & 0.3333\\ -0.3333 & -0.6667 & 0.3333 \end{array}\right]$$

**Figure 5.**
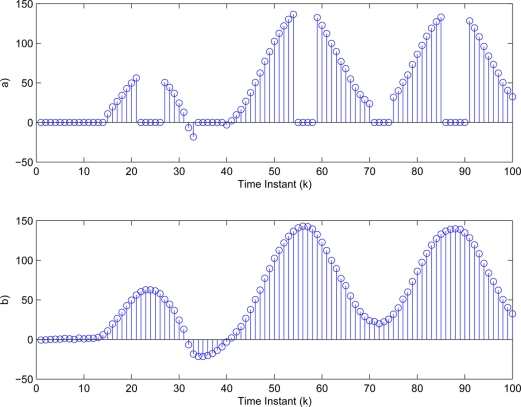
Transmitted measurements of sensor $y_{k}^{1}$ by **(a)** event-triggered and **(b)** time-triggered.

**Figure 6.**
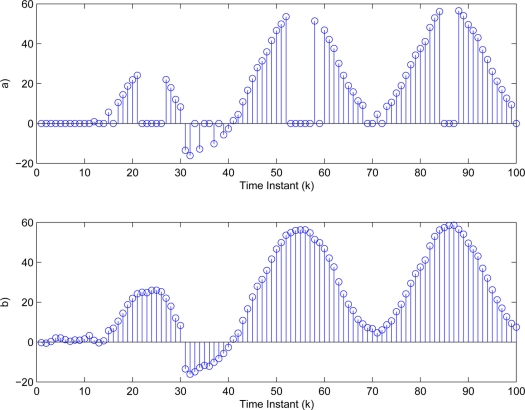
Transmitted measurements of sensor $y_{k}^{2}$ by **(a)** event-triggered and **(b)** time-triggered.

**Figure 7.**
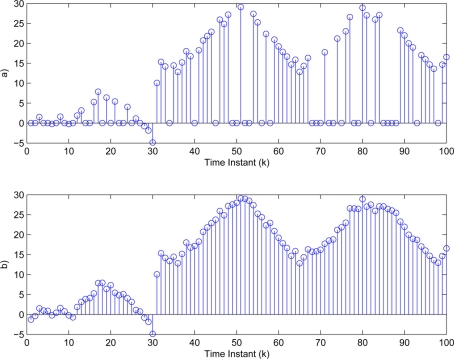
Transmitted measurements of sensor $y_{k}^{3}$ by **(a)** event-triggered and **(b)** time-triggered.

Furthermore, we choose $\frac{1}{30}$-fold of the maximal amplitude of $y_{k}^{i}$ (*i* = 1,2,3). Then, the threshold values in Equation (3) are determined as δ_1_ = 4.8406, δ_2_ = 2.0382, δ_3_ = 1.0009. The sufficiently small threshold ε*_i_* (*i* = 1,2,3) in Equation (7) is given as 10^−4^. Then, Figure 5(a)–7(a) show all the transmitted measurements of sensors with event-triggered scheme. Figures 5(b)–7(b) indicate all the transmitted measurements of sensors with time-triggered scheme. By comparison, the sensors with event-triggered scheme transmit only 66%, 67%, and 58% of samples produced by time-triggered scheme, respectively. In other words, the resource utilization by the event-triggered scheme can be obtained 34%, 33% and 42% improvement, respectively.

**Figure 8.**
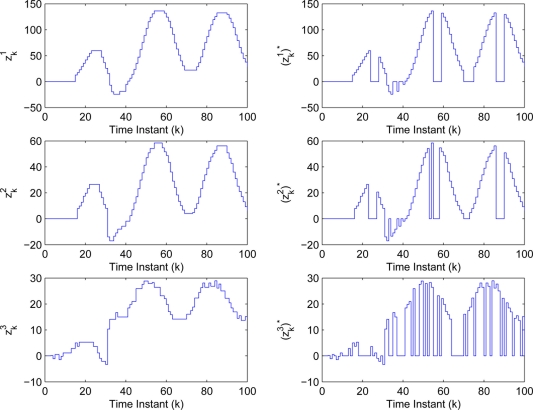
Measurements **(a)** with compensation and **(b)** without compensation.

**Figure 9.**
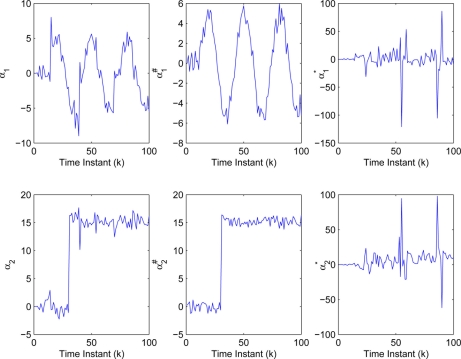
Innovation sequences of residual α*_k_*, $\alpha_{k}^{\#}$ and $\alpha_{k}^{★}$.

The measurements used in the simulation are indicated in Figure 8, where $z_{k}^{i}$ (*i* = 1,2,3) and ${\left(z_{k}^{i}\right)}^{★}$ (*i* = 1,2,3) represent the measurements with compensating and without compensating some values for the unsent ones, respectively. By Algorithm 1 and the measurements $z_{k}^{i}$, a modified FIF is implemented to the NCS with event-triggered sampling. Figure 9 shows the innovation sequences of residual $\alpha_{k}={\left[\begin{array}{cc} {\left(\alpha_{1}\right)}_{k} & {\left(\alpha_{2}\right)}_{k} \end{array}\right]}^{T}$, $\alpha_{k}^{\#}={\left[\begin{array}{cc} {\left(\alpha_{1}^{\#}\right)}_{k} & {\left(\alpha_{2}^{\#}\right)}_{k} \end{array}\right]}^{T}$ and $\alpha_{k}^{★}={\left[\begin{array}{cc} {\left(\alpha_{1}^{★}\right)}_{k} & {\left(\alpha_{2}^{★}\right)}_{k} \end{array}\right]}^{T}$, which correspond to the residual obtained by the modified FIF described in Algorithm 1, the Keller’s FIF in [27] using the time-triggered samples and the Keller’s FIF using the measurements ${\left(z_{k}^{i}\right)}^{★}$, (*i* = 1,2,3), respectively.

In order to compare the simulation results obtained by different schemes, we propose the root mean square (RMS) of the fault estimation errors as a performance index. This error for the scalar variable *n_i_* with respect to its estimate α*_i_* for *N_s_* simulation steps is defined as

(17) $${\mathrm{RMS}}_{i}≜\sqrt{\frac{\sum_{j=1}^{N_{s}}{\left[n_{i}^{j}-\alpha_{i}^{j}\right]}^{2}}{N_{s}}}$$

where $n_{i}^{j}$ is the value of the variable *n_i_* in the *j*th step, $\alpha_{i}^{j}$ is the estimate of $n_{i}^{j}$ and *i* = 1,2. Table 1 indicates the RMS of the fault estimation errors by different methods.

From Figure 9 and Table 1, the performance of the modified FIF using less sensor data transmission experiences graceful degradation in contrast to the Keller’s FIF using time-triggered samples. By graceful degradation, it means that a system degenerates in such a manner that it continues to operate, but provides a reduced level of service rather than causing total breakdown. On the other hand, the Keller’s FIF using the measurements ${\left(z_{k}^{i}\right)}^{★}$ (*i* = 1,2,3) fails to work completely, while the number of the total sensor data transmission is same as the modified FIF used. From these results, we can draw a conclusion that the modified FIF has an advantage in the tradeoff between communication cost and fault estimation performance.

**Figure 10.**
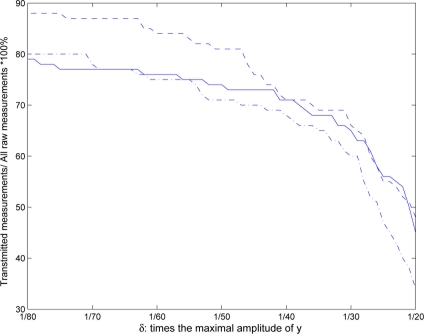
Percentage of transmitted samples to total samples in relation to *δ_i_* by event-triggered scheme.

In order to establish the relationships between the number of the sensor data transmissions, the RMS of the fault estimation errors by different methods and the threshold values δ*_i_* (*i* = 1,2,3), some further simulations are done by adjusting the threshold values *δ_i_* (*i* = 1,2,3) from 1/20 to 1/80 of the maximal amplitude of $y_{k}^{i}$ (*i* = 1,2,3). Figure 10 shows the percentage of transmitted samples to total samples in relation to *δ_i_* by event-triggered scheme, wherein the real line, dash line and dash-dotted line represent the percentage for sensor $y_{k}^{1}$, $y_{k}^{2}$ and $y_{k}^{3}$, respectively. From Figure 10, it can be seen that the sensor data transmission rate is inversely proportional to *δ_i_*, namely the communication cost reduced by the event-triggered scheme will increase when *δ_i_* increases, and vice versa.

**Figure 11.**
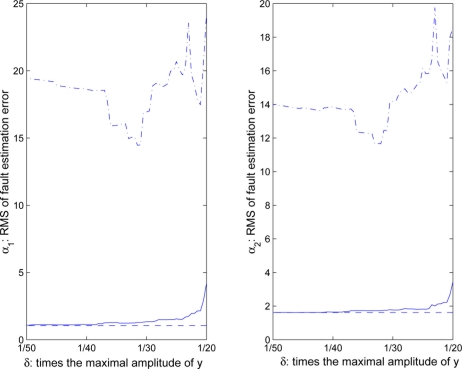
RMS of fault detection errors in relation to *δ_i_*.

In Figure 11, the real line, dash line and dash-dotted line represent the fault estimation performance of the modified FIF, the Keller’s FIF with time-triggered sampling and the FIF with the measurements ${\left(z_{k}^{i}\right)}^{★}$, respectively. It can be seen that the fault estimation performance of the modified FIF is improved and eventually approaches the performance of Keller’s FIF with time-triggered sampling as *δ_i_* decreases. However, the performance of the FIF with the measurement ${\left(z_{k}^{i}\right)}^{★}$ (*i* = 1,2,3) is very poor even by flexibly adjusting the threshold *δ_i_*.

## 5. Conclusions

This paper is concerned with the implementation problem of fault isolation filter for networked control system with the send-on-delta scheme which is one of the event-triggered sampling strategies. By send-on-delta, the sensor data is transmitted only when the absolute value of difference between the current sensor value and the previously transmitted one is greater than the given threshold value. Based on this scheme, a modified fault isolation filter for a discrete-time networked control system with multiple faults is then implemented by a particular form of the Kalman filter. In contrast to the Keller’s fault isolation filter using time-triggered samples, the proposed fault isolation filter improves the resource utilization with graceful fault estimation performance degradation. Also, we can improve the performance of the modified FIF by flexibly adjusting the threshold values with taking resource utilization into account.

Throughout the paper, no network-induced packet losses are taken into account in the model of the networked control system. Further study of the fault isolation filter for networked control system with event-triggered sampling and packet losses is encouraged.

## Figures and Tables

**Table 1.** RMS of the fault estimation errors.

*α*	(*α*_1_)*_k_*	$\left(\alpha_{1}^{\#}\right)k$	$\left(\alpha_{1}^{★}\right)k$	(*α*_2_)*_k_*	$\left(\alpha_{2}^{\#}\right)k$	$\left(\alpha_{2}^{★}\right)k$
RMS	1.6258	0.9368	18.0693	1.7998	1.6235	14.2398
